# Reabsorption cross section of Nd^3+^-doped quasi-three-level lasers

**DOI:** 10.1038/s41598-019-42012-4

**Published:** 2019-04-04

**Authors:** Fei Chen, Junjie Sun, Renpeng Yan, Xin Yu

**Affiliations:** 1 State Key Laboratory of Laser Interaction with Matter, Innovation Laboratory of Electro-Optical Technology, Changchun Institute of Optics, Fine Mechanics and Physics, Chinese Academy of Sciences, Changchun, 130033 China; 2 National Key Laboratory of Science and Technology on Tunable Laser, Harbin Institute of Technology, Harbin, 150080 China

## Abstract

The ^4^*F*_*3/2*_ → ^4^*I*_*9/2*_ laser transition of Nd^3+^-doped crystals emitting at 900 nm is a standard quasi-three-level laser system. The reabsorption effect is one of the factors that restricts laser output power. Based on rate equations, a theoretical model considering the reabsorption effect for continuous-wave Nd^3+^-doped quasi-three-level lasers is established. The simulation results indicate that the reabsorption effect should be restrained to improve laser characteristics, which are mainly influenced by the Nd^3+^-doping concentration, laser medium length, pumping beam divergence angle and output mirror transmissivity. The optimal experimental results illustrate the availability of a theoretical model that considers the reabsorption effect. To quantitatively evaluate the reabsorption effect of a Nd^3+^-doped laser medium, a reabsorption cross section is proposed for the first time to the best of our knowledge. Comparing the experimental results and theoretical calculation results, the reabsorption cross section is estimated for a 912-nm Nd:GdVO_4_ laser, 914-nm Nd:YVO_4_ laser and 946-nm Nd:YAG laser.

## Introduction

High-power Nd^3+^-doped lasers operating at approximately 900 nm have been extensively studied because of their unique applications. First, 900-nm lasers with high power are efficient sources that can pump Yb-doped fibers and crystals to obtain a laser at approximately 980 nm^1^. Second, these lasers are used for ozone measurements and remote sensing in water-vapor lidars and differential-absorption lidars. Moreover, these lasers are capable of generating blue lasers by frequency doubling^2–5^. Many studies have been performed to achieve high-power Nd^3+^-doped lasers by employing various media and crystals, including Nd:YAG, Nd:YVO_4_ and Nd:GdVO_4_. In 1987, a 946-nm Nd:YAG laser was demonstrated for the first time^6^. Zhou *et al*. demonstrated a 8.3-W laser at 946 nm using a Nd:YAG rod in 2005^7^. In the same year, J. Gao realized a 25.4-W laser at 946 nm with a thin disk Nd:YAG crystal^8^. In 2017, Y. Sun reported a 946-nm Nd:YAG Q-switched laser with an achieved pulse energy of 2.63 mJ with a 10.8-ns pulse width and a peak power of approximately 244 kW^9^. A 914-nm laser in a Nd:YVO_4_ crystal was reported by P. Zeller and P. Peuser in 2000 with a maximum output power of 3.0 W and a slope efficiency of 22.8%^10^. In 2009, W. Gong reported a V-shaped cavity that emitted a 7.3-W laser at 914 nm^11^. In 2016, P. Jiang demonstrated an efficient 914-nm Nd:YVO_4_ laser under double-end polarized pumping, and a 17.7-W continuous wave output was obtained with an efficiency of 33.4%^12^. In a Nd:GdVO_4_ crystal, a 912-nm laser with an output power of 8.0 W was achieved by X. Yu *et al*. in 2008^13^. In 2009, Y. F. Lv reported a Nd:GdVO_4_ laser with an optical efficiency of 58.2% and a slope efficiency of 67.9%, corresponding to an output power of 8.1 W^14^. Afterwards, a novel indium-solder technology was demonstrated by R. P. Yan to improve the performance of a 912-nm Nd:GdVO_4_ laser^15^.

The operation of 900-nm lasers in Nd^3+^-doped crystals is a standard quasi-three-level laser system because the lowest laser level is the uppermost component of the five crystal-field components of the ground-state ^4^*I*_*9/2*_ level. A quasi-three-level laser system is different from the traditional four-level and three-level laser systems but has the characteristics of both. Achieving a high-power 900-nm laser in Nd^3+^-doped crystals is challenging. The main challenge can be ascribed to the nature of a quasi-three-level laser. On the one hand, the stimulated-emission cross section of the transition is small. On the other hand, the thermal population on the lowest laser level at room temperature will lead to a significant reabsorption effect^16^. The concept of the reabsorption effect in quasi-three-level lasers was proposed by T. Y. Fan and R. L. Byer in 1987^6^. W. P. Risk modeled longitudinally pumped solid-state lasers, including reabsorption loss, in 1988^16^, and this model could be employed to analyze laser characteristics, such as threshold and slope efficiency. Since 1988, the reabsorption effect has attracted more attention^17,18^. A quasi-three-level laser output performance is seriously influenced by the reabsorption effect. Therefore, a method to quantitatively evaluate the reabsorption effect is indispensable for completing quasi-three-level laser theory.

In this paper, a reabsorption cross section for evaluating the reabsorption effect of Nd^3+^-doped quasi-three-level lasers is proposed for the first time. Theoretical calculations considering the reabsorption effect suggest that the output performance is mainly influenced by the Nd^3+^-doping concentration, laser medium length, pumping beam divergence angle and output mirror transmissivity. In this experiment, Nd^3+^-doped quasi-three-level lasers using different Nd^3+^-doped laser media with high output power are realized. The experimental results correspond well to the simulation results considering the reabsorption effect. The numerical values of the reabsorption cross sections of a 912-nm Nd:GdVO_4_ laser, 914-nm Nd:YVO_4_ laser and 946-nm Nd:YAG laser were determined by comparing the theoretical and experimental results.

## Results

### Theoretical model

**Figure 1:**
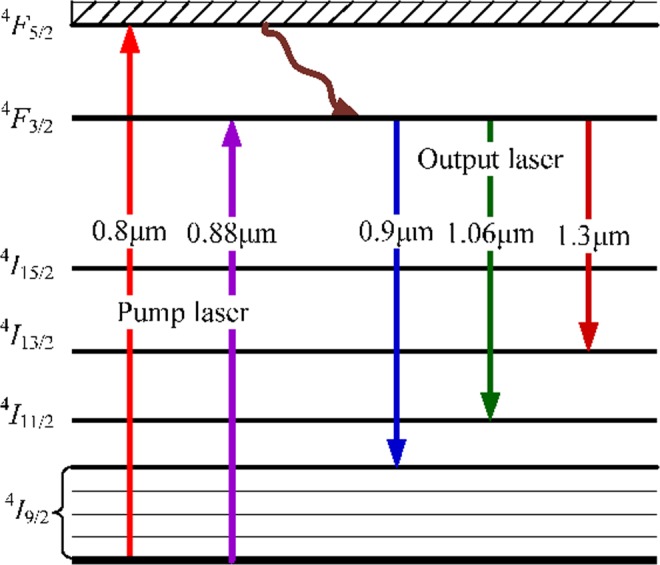
Energy-level diagram and laser emissions of Nd^3+^ crystals.

Figure 1 shows the energy levels and laser emissions of Nd^3+^ crystals. Nd^3+^ crystals with a large absorption cross section correspond to pump laser wavelengths of 800 nm and 880 nm. Laser output of approximately 1060 nm and 1300 nm operates between ^4^*F*_*3/2*_ → ^4^*I*_*11/2*_ and ^4^*F*_*3/2*_ → ^4^*I*_*13/2*_, respectively. The other transition ^4^*F*_*3/2*_ → ^4^*I*_*9/2*_ is a quasi-three-level transition, and the lowest level is the uppermost level of the five branches of the ground state ^4^*I*_*9/2*_ energy level. For example, a laser operating at 912 nm is a quasi-three-level laser system, and the wave number of the lower level is 408 cm^−1^. Assuming the number of thermal population particles of ^4^*I*_*9/2*_ level is *N*_*L*_, and the ratio of the particles number of Z_5_ branches to the total number in the ground state is *f*_1_. Therefore, the number of particles occupying the lower thermal level is *N*_1_ = *f*_1_*N*_*L*_. Similarly, the thermal population particles number of ^4^*F*_*3/*2_ is *N*_*U*_, and the proportion of the particles number under the branch is *f*_2_, thus, the particles number in the upper thermal level is *N*_2_ = *f*_*2*_*N*_*U*_.

**Figure 2:**
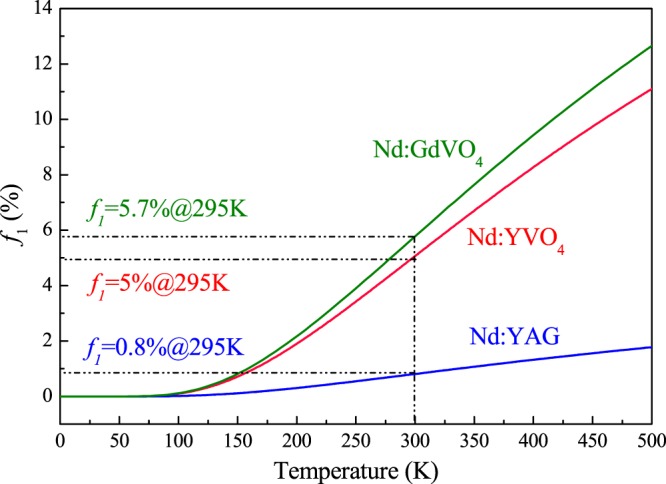
*f*_1_ versus the working temperature of the laser medium.

The relationship between *f*_1_ and the operating temperature of the laser medium for Nd:GdVO_4_, Nd:YVO_4_ and Nd:YAG is shown in Fig. 2. At room temperature, the ratio of the number of thermal population particles on the Z_5_ sublevel of the total number of ground states in Nd:GdVO_4_, Nd:YVO_4_ and Nd:YAG is 5.7%, 5% and 0.8%, respectively. The difference is caused by the different wave numbers of the lowest levels, i.e., 408 cm^−1^, 433 cm^−1^ and 857 cm^−1^, respectively. Figure 2 shows that the number of particles, *f*_1_, in the quasi-three-level laser system linearly increases as the temperature increases. The laser reabsorption effect on the ^4^*F*_*3/2*_ → ^4^*I*_*9/2*_ level transition will influence laser characteristics, such as the threshold power and output slope efficiency of a laser. Thus, lasers need to operate at temperatures as low as possible.

The analysis of the performance of a Nd^3+^-doped quasi-three-level laser considering the reabsorption effect begins with the rate equation. Pumping and oscillating lasers are assumed to be circularly symmetric TEM_00_ Gaussian beams. Supposing that the density of inverse particles is ${\rm{\Delta }}N(r,z)={N}_{2}(r,z)-{N}_{1}(r,z),$ Equation (1) describes the density change of the upper and lower energy levels of Nd^3+^-doped lasers, where ${\rm{\Delta }}{N}^{0}={N}_{2}^{0}-{N}_{1}^{0}$ is the pump under the action of the inverse particle density number, *τ* stands for the laser-level life, *c* is the speed of light in a vacuum, *σ* is the laser-stimulated emission cross section, *n* is the laser refractive index of the medium, *z* is a coordinate in the direction of the laser axis, and *r* is a radial coordinate.

1 $$\frac{d{\rm{\Delta }}N(r,z)}{dt}=({f}_{1}+{f}_{2}){R}_{p}{r}_{p}(r,z)-\frac{{\rm{\Delta }}N(r,z)-{\rm{\Delta }}{N}^{0}}{\tau }-\frac{({f}_{1}+{f}_{2})c\sigma {\rm{\Delta }}N(r,z)}{n}\Phi \varphi (r,z)=0$$

*R*_*p*_ represents the rate at which particles are emitted to the upper level in the pump and can be expressed as ${R}_{p}={P}_{in}\eta /h{\nu }_{P}$, where *P*_*in*_ is the incident pump power,$\eta =1-\exp \,(-\,\alpha l)$ is the absorption rate of the laser medium with a length of *l* and absorption coefficient of *α*, *h* is the Planck constant and *v*_*p*_ is the frequency of the pumping light. The number of photons in the laser cavity can be expressed as ${\rm{\Phi }}=2nl{P}_{L}/ch{\nu }_{L}$, where *P*_*L*_ is the dynamic laser power in one direction of the cavity and *v*_*L*_ is the laser frequency. Equation (2) presents the number of reversed particles above the threshold, where ${f}^{\ast }={f}_{1}+{f}_{2}$.

2 $${\rm{\Delta }}N(r,z)=\frac{\tau {f}^{\ast }{R}_{p}{r}_{P}(r,z)-{N}_{1}^{0}}{1+\frac{c\sigma \tau }{n}{f}^{\ast }{\rm{\Phi }}\varphi (r,z)}$$

For the analysis of a Nd^3+^-doped laser, the parameters were defined as follows. $a={\omega }_{P}/{\omega }_{l}$ is the ratio of the waist radius of the pump beam to the waist radius of the oscillating beam, and $B=2{N}_{1}^{0}\sigma l/(L+T)$ is the ratio of the reabsorption loss to the intrinsic loss of the laser cavity. $F=4{P}_{in}\tau \sigma \eta /{\rm{\pi }}h{\nu }_{P}{\omega }_{L}^{2}(L+T)$ is a parameter proportional to the incident pump power, and $S=2c\sigma \tau {\rm{\Phi }}/n\pi {\omega }_{L}^{2}l$ is a parameter proportional to the power of the lasing laser.*ω*_*P*_ and *ω*_*L*_ are the waist radii of the pump beam and the laser beam, respectively. *L* is the loss of one round-trip of the cavity, and *T* is the transmissivity of the output mirror. Therefore, the slope efficiency, *dP*_*out*_*/dP*_*in*_, of the output laser light can be expressed as

3 $$\frac{d{P}_{out}}{d{P}_{in}}=\frac{T{\nu }_{L}\eta }{(L+T){\nu }_{P}{({f}_{1}+{f}_{2})}^{2}{F}^{2}}\frac{1+\frac{B}{({f}_{1}+{f}_{2})S}\,\mathrm{ln}[1+({f}_{1}+{f}_{2})S]}{{\int }_{0}^{\infty }\frac{[\exp (-x)-\frac{B{a}^{2}}{({f}_{1}+{f}_{2})F}]\exp (-2{a}^{2}x)}{{[1+({f}_{1}+{f}_{2})S\exp (-{a}^{2}x)]}^{2}}dx}$$

where $x=2{r}^{2}/{\omega }_{P}^{2}$. From Equation (3), we can obtain the numerical relationship between the output power of a Nd^3+^-doped quasi-three-level laser and the pump power. The theoretical analysis shows that reabsorption has a remarkable influence on the output power and slope efficiency of Nd^3+^-doped quasi-three-level lasers^19^.

### Simulations and analysis

According to the theoretical model considering the reabsorption effect, calculated results for the Nd:GdVO_4_ laser, Nd:YVO_4_ laser and Nd:YAG laser were obtained employing the parameters in Table 1.

**Table 1:** Main parameters of Nd^3+^-doped quasi-three-level lasers.

Parameter	Nd:GdVO_4_	Nd:YVO_4_	Nd:YAG
*f*_1_ Fraction of the population density in the lowest laser level	0.5050	0.5055	0.51
*f*_2_ Fraction of the population density in the upper laser level	0.0534	0.049	0.04
*n* Refractive index	1.973	1.973	1.816
*τ* Lifetime of the upper laser level	90	100	230
*α* Absorption coefficient	1.53	1.53	2.95
*σ* Stimulated-emission cross section	6.6 × 10^−20^	4.8 × 10^−20^	3.7 × 10^−20^
*ω*_*p*_ Waist radius of the pump beam	200	200	200
*ω*_*l*_ Waist radius of the laser beam	200	200	200

#### 912-nm Nd:GdVO_4_ laser calculation

**Figure 3:**
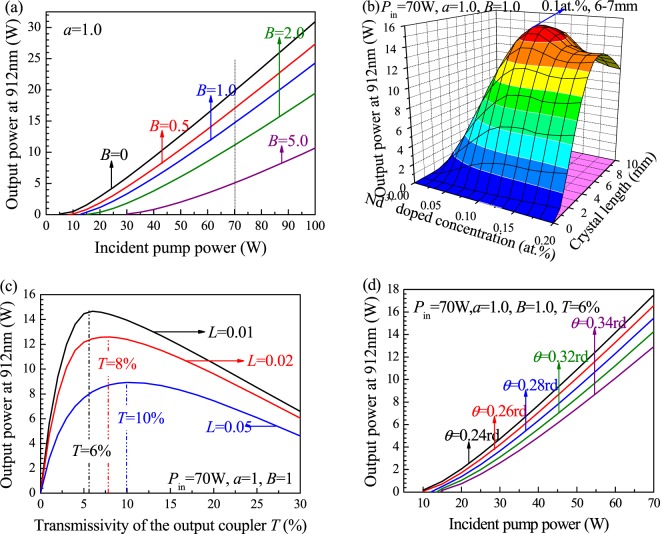
Output power of a 912-nm Nd:GdVO_4_ laser versus (**a**) the pump power with different *B*. (**b**) Nd^3+^-doping concentration and length of the laser medium. (**c**) Transmissivity of the output coupler. (**d**) Incident pump power at different *θ* values.

The calculation results of the relationship between the output power of a 912-nm Nd:GdVO_4_ laser with different parameters are depicted in Fig. 3. Figure 3(a) shows the output power as a function of the incident pump power considering different reabsorption effects, and the ratio of the waist radius of the pump beam to the laser beam is *a* = 1.0. The output power of the 912-nm laser is strongly influenced by the reabsorption effect. The output power decreases from 19.8 W to 5.08 W as *B* increases from 0 to 5.0 at a pump power of 70 W.

An optimum doping concentration and crystal length can improve laser characteristics. Additionally, the output power and slope efficiency of a laser are influenced by these parameters. Figure 3(b) shows the calculated results for the output power versus the doping concentration and crystal length. The Nd:GdVO_4_ laser crystal with a doping concentration of 0.1 at.% and a length of 6–7 mm is optimal to obtain a 912 nm laser with power up to 14.7 W.

Using the above optimization parameters, the relationship between the output power and the transmissivity of the output mirror, *T*, is shown in Fig. 3(c). When *L* = 0.01, the optimum transmissivity of the output mirror is approximately 6%. When *L* = 0.05, the optimum transmissivity of the output mirror is approximately 10%. With an increase in *L*, the laser output power rapidly decreases because the laser emission cross section of a 912-nm quasi-three-level system decreases, leading to low laser gain. Additionally, the output power is greatly influenced by cavity loss. Therefore, to achieve high efficiency output from a 912-nm laser, the coating quality of the resonator mirror must be improved and the cavity loss must be reduced as much as possible. Figure 3(d) shows the output power versus the pump power at different divergence angles, *θ*. As the divergence angle, *θ*, decreases, the output power of the laser increases at the same injection pump power. Thus, the lens focal length used for the collimated focusing coupling system needs to be optimized to further increase the laser output power.

#### 914-nm Nd:YVO_4_ laser calculation

**Figure 4:**
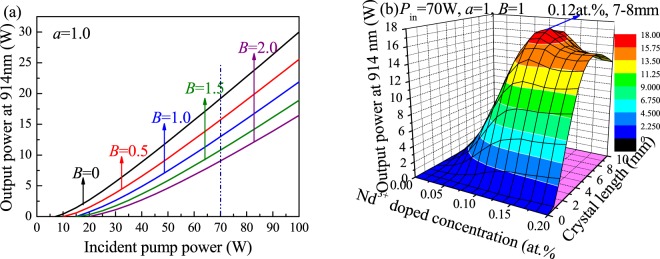
Output power of a 914-nm Nd:YVO_4_ laser versus (**a**) the pump power with different *B*. (**b**) Nd^3+^-doping concentration and length of the laser medium.

Similar to the 912-nm Nd:GdVO_4_ laser system, the transition of the 914-nm Nd:YVO_4_ laser belongs to a quasi-three-level laser system. The difference is that the wave number of the energy level in a 914-nm laser is 433 cm^−1^. According to the theoretical model, the output power of a 914-nm Nd:YVO_4_ laser versus different parameters was calculated. Figure 4(a) shows the 914-nm laser output performance considering different reabsorption effects at *a* = 1.0. When *P*_*in*_ = 70 W, the maximum output power of the continuous laser decreases from 19.2 W to 9.05 W as *B* increases from 0 to 5.0. Figure 4(b) shows the relationship between the laser output power at 914 nm and the doping concentration and length of the laser medium at *P*_*in*_ = 70 W. A Nd:YVO_4_ laser with a doping concentration of 0.12 at.% and a length of 7–8 mm is optimal and can obtain a maximum output power of 17.2 W at 914 nm.

#### 946-nm Nd:YAG laser calculation

**Figure 5:**
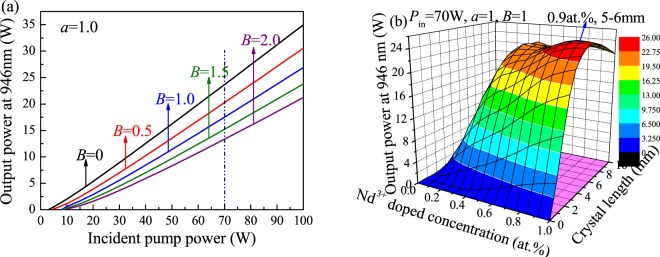
Output power of a 946-nm Nd:YAG laser versus (**a**) the pump power with different *B*. (**b**) Nd^3+^-doping concentration and length of the laser medium.

The laser input-output curves of a 946-nm Nd:YAG laser considering different reabsorption effects are shown in Fig. 5(a). When *P*_*in*_ = 70 W, the maximum output power of the 946-nm continuous-wave laser decreases from 23.7 W to 13.3 W as *B* increases from 0 to 5.0. Figure 5(b) shows the relationship between the output power and the doping concentration and the length of the laser medium. The results suggest that a Nd:YAG laser crystal with a doping concentration of 0.9 at.% and a length of 5–6 mm can be used to realize a high-power-output 946-nm laser.

### Experimental setup

**Figure 6:**
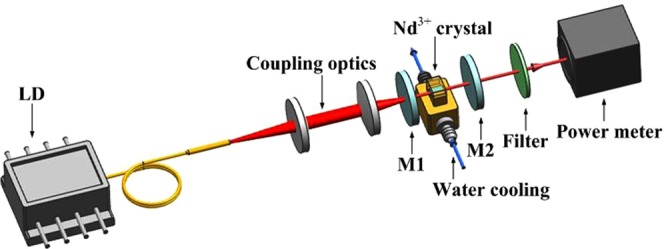
Diagram of the experimental setup for the quasi-three-level Nd^3+^-doped laser system.

To analyze the reabsorption effects of 912-nm Nd:GdVO_4_ laser, 914-nm Nd:YVO_4_ laser and 946-nm Nd:YAG laser, a diode-end-pumped quasi-three-level Nd^3+^-doped laser was employed, as shown in Fig. 6. The output light of the 808 nm LD (nLIGHT, Inc.) was coupled into the laser medium by a collimating focusing optical system, and the maximum output power of LD is 110 W. The laser media were provided by Castech Inc, and the surface of the crystal was coated with an antireflection film at 808–1340 nm. The crystals were mounted in a copper micro channel heat sink and maintained at 10 °C by water cooling. The reflecting mirror M1 was coated with a highly reflective film at approximately 900 nm (HR > 99.9%) and a high-transmissivity film at 808 nm (HT, T > 98%). The output mirror M2 partially transmitted at approximately 900 nm. To suppress the generation of four-level-laser parasitic oscillations, all mirrors were coated with a high-transmissivity film at 1063 nm and 1340 nm. The specifications of the filter are as follows: HT @ 900 nm (T > 95%), HR @ 808 nm, 1063 nm (T < 1%). The model of the power meter was PM30 (Coherent Inc., USA) with a response range of 0.19–11 μm and a maximum range and resolution of 50 W and 10 mW, respectively. For each crystal, the system was optimized with various parameters according to the theoretical calculations. Afterwards, the reabsorption cross section was determined by comparing the optimal results and the simulation results.

### Evaluation of the reabsorption cross section

#### 912-nm Nd:GdVO_4_ laser

The high power output of the 912-nm Nd:GdVO_4_ laser was obtained by optimizing parameters, which has been reported in Ref.^19^. The transmissivity of the output mirror was 6%; the doping concentration and length of the laser medium were 0.1 at.% and 6 mm, respectively. The output performance of 912-nm Nd:GdVO_4_ laser was influenced by the spatial distribution of pump beam and laser beam in the crystal. The laser beam waist was fixed after optimizing the cavity structure. Afterwards, the beam waist and the divergence angle of pump beam in the crystal were optimized by designing the coupling optics. The optimum parameters were as follows. The focal length of the collimation lens was 21.3 mm; and the ratio of the waist radius of the pump beam to the laser beam was *a* = 1.0. A maximum output power of 16.2 W was obtained with an optical conversion efficiency of 24.2% and an average slope efficiency of 41.7%. The experimental results are in good agreement with the theoretical calculation results, and the agreement verifies the validity of the theoretical simulation considering the reabsorption effect.

Therefore, to evaluate the reabsorption effect in Nd^3+^-doped laser media, the reabsorption cross section, *σ*_*r*_, was defined and deduced from the excited emission cross section of the original rate equation. By comparing the theoretical calculation for the 912-nm laser output and the experimental results, the value of the reabsorption cross section, *σ*_*r*_, of the 912-nm Nd:GdVO_4_ laser was estimated.

**Figure 7:**
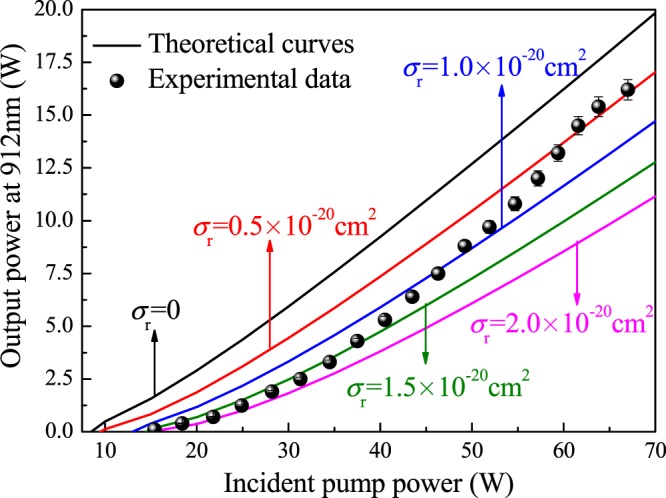
Comparison of the experimental results and the calculated curves of a 912-nm Nd:GdVO_4_ laser.

As shown in Fig. 7, the input-output curves of the 912-nm Nd:GdVO_4_ laser for different reabsorption cross sections were selected and compared with the experimentally measured values. It can be clearly seen that if the reabsorption effect is not taken into account, the output power curve greatly differs from the measured value. The laser threshold increases drastically due to the reabsorption effect. The reabsorption cross section of the 912-nm Nd:GdVO_4_ laser was estimated to be *σ*_*r*_ = (1.0 ± 0.5) × 10^−20^ cm^2^.

The reabsorption effect is relevant to the number of particles on the lower energy level, and is dependent to the absorption of particles by pump laser. There are many particles being absorbed while the pump power is low, corresponding large reabsorption cross section, which leads high laser threshold. And the laser is unstable under low pump power, thus the rate of output power increasing is slow. While the pump power is high, the laser operation is stable. The rate of output power increasing gets fast. And there are less particles being absorbed by pump laser, corresponding to small reabsorption cross section.

#### 914-nm Nd:YVO4 laser

**Figure 8:**
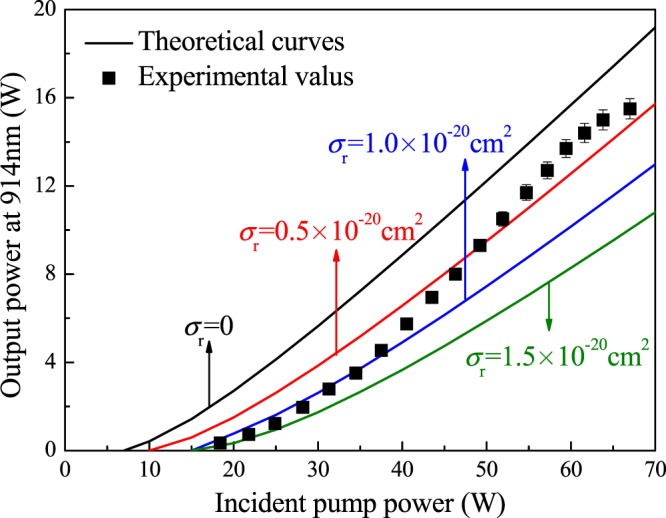
Comparison between the experimental results and the calculated curves for the 914-nm Nd:YVO_4_ laser.

The optimization of the 914-nm Nd:YVO_4_ laser system was similar to that of the 912-nm Nd:GdVO_4_ laser system. Parameters, including the Nd^3+^-doping concentration, the length of the laser medium and the transmissivity of the output mirror, were optimized according to the theoretical calculation. As ref.^20^ reported, the optimum length of the Nd:YVO_4_ crystal is 6 mm with a Nd^3+^-doping concentration of 0.1 at.%, and the optimal transmissivity of the output mirror is *T* = 6%. With an incident pump power of 67 W, an up to 15.5 W continuous wave 914-nm laser was obtained with an average slope efficiency of 37.6%. The calculated results were compared with the experimental results, as shown in Fig. 8. The reabsorption cross section of the 914-nm Nd:YVO_4_ laser was estimated to be *σ*_*r*_ = (0.5 ± 0.5) × 10^−20^ cm^2^. This value is smaller than that of the Nd:GdVO_4_ laser because the fraction of the population density in the lowest laser level of the Nd:GdVO_4_ laser is greater, leading to a more serious reabsorption effect. The variation of reabsorption cross section with pump power is similar as 912-nm Nd:GdVO_4_ laser.

#### 946-nm Nd:YAG laser

**Figure 9:**
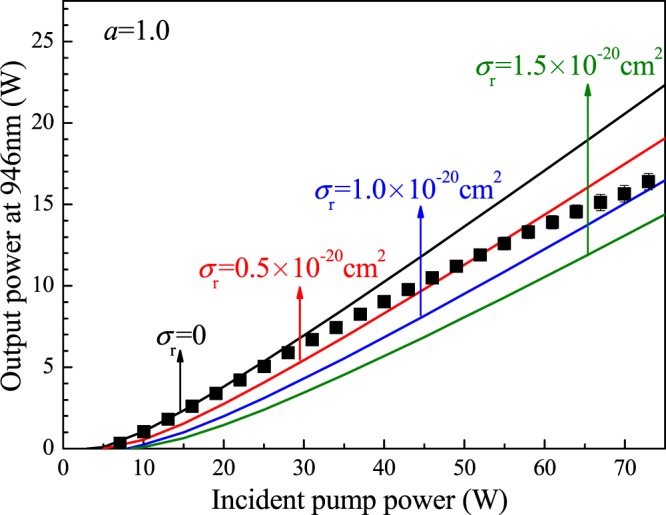
Comparison between the experimental results and the calculated curves for the 946-nm Nd:YAG laser.

Similarly, the 946-nm Nd:YAG laser was optimized as presented in ref.^21^. A 5-mm-length, 0.3-at.% Nd:YAG crystal was employed in the experiment, which deviated from the theoretical result. The reason for the deviation was the high absorption in the pump front end when a laser medium with a high doping concentration was used. When the 0.3-at.%, 5-mm-length laser medium was employed, the output power and average slope efficiency were not the highest, but the laser output power grew linearly with the pump power. Furthermore, using the transmissivity, *T* = 9%, output coupler, a maximum output power of 17.2 W was obtained with a slope efficiency of 26.5%. The experimental results for the 946-nm Nd:YAG laser system were compared with the theoretical input-output curves, as shown in Fig. 9. In the experiment, the slope of the input-output curve decreased when the input power was greater than 50 W, which was inconsistent with the theoretical results. This inconsistency is ascribed to the use of a laser medium with a high doping concentration. The high absorption efficiency leads that the output laser tends to be saturated under high pump power. The reabsorption cross section of the 946-nm Nd:YAG laser is approximately *σ*_*r*_ = (0.5 ± 0.5) × 10^−20^ cm^2^.

## Discussion

A reabsorption cross section was proposed to evaluate the reabsorption effect in a Nd^3+^-doped quasi-three-level system. Theoretical and experimental investigations of Nd^3+^-doped lasers were presented to determine the value of the reabsorption cross section. The effect of parameters, such as the transmissivity of the output mirror, doping concentration and length of laser medium, on a 912-nm Nd:GdVO_4_ laser, 914-nm Nd:YVO_4_ laser and 946-nm Nd:YAG laser was analyzed and theoretically calculated. In the experiments, the output power as a function of the input power was obtained using the optimal parameters, and the results were consistent with the theoretical calculation results considering the reabsorption effect. The reabsorption cross section of the 912-nm Nd:GdVO_4_ laser was estimated to be (1.0 ± 0.5) × 10^−20^ cm^2^ and was estimated to be (0.5 ± 0.5) × 10^−20^ cm^2^ for both the 914-nm Nd:YVO_4_ laser and 946-nm Nd:YAG laser.

## Methods

The analysis of the performance of Nd^3+^-doped quasi-three-level laser systems considering the reabsorption effect began with the rate equation. Parameters such as the doping concentration, length of the laser medium, transmissivity of the output mirror, cavity loss and divergence angle were considered to analyze how they influence the output power, threshold and slope efficiency of a laser. According to the theoretical analysis, simulations were performed to obtain the theoretical optimal parameters. Afterwards, the parameters were experimentally optimized, and the experimental results corresponded to the theoretical results. Finally, by comparing the theoretical results and optimal experimental results, the value of the reabsorption cross section was estimated.
